# Supplementary material for: Formin-2 drives polymerisation of actin filaments enabling segregation of apicoplasts and cytokinesis in Plasmodium falciparum
Source: eLife. 2019 Jul 19;8:e49030. doi: 10.7554/eLife.49030 (PMC6688858; doi:10.7554/eLife.49030)
Supplement: Figure 1—source data 1. [file elife-49030-fig1-data1.docx]

Figure 1-source data 1

(Source data for table in Figure 1A)

| **Effect of conditional knock out of *actin-1* on:** | ***Plasmodium falciparum*** | ***Toxoplasma gondii*** |
| --- | --- | --- |
| **Apicoplast segregation** | **+++**  **(Das, Lemgruber et al. 2017)** | **+++**  **(Andenmatten, Egarter et al. 2013, Egarter, Andenmatten et al. 2014, Whitelaw, Latorre-Barragan et al. 2017)** |
| **Cytokinesis/daughter formation** | **++**  **(Das, Lemgruber et al. 2017)** | **-** |
| **Invasion into host cell** | **+++**  **(Das, Lemgruber et al. 2017)** | **++**  **(Andenmatten, Egarter et al. 2013, Egarter, Andenmatten et al. 2014, Whitelaw, Latorre-Barragan et al. 2017)** |
| **Egress from host cell** | **-**  **(Das, Lemgruber et al. 2017)** | **+++**  **(Andenmatten, Egarter et al. 2013, Whitelaw, Latorre-Barragan et al. 2017)** |

Andenmatten, N., S. Egarter, A. J. Jackson, N. Jullien, J. P. Herman and M. Meissner (2013). "Conditional genome engineering in Toxoplasma gondii uncovers alternative invasion mechanisms." Nat Methods **10**(2): 125-127.

Das, S., L. Lemgruber, C. L. Tay, J. Baum and M. Meissner (2017). "Multiple essential functions of Plasmodium falciparum actin-1 during malaria blood-stage development." BMC Biol **15**(1): 70.

Egarter, S., N. Andenmatten, A. J. Jackson, J. A. Whitelaw, G. Pall, J. A. Black, D. J. Ferguson, I. Tardieux, A. Mogilner and M. Meissner (2014). "The toxoplasma Acto-MyoA motor complex is important but not essential for gliding motility and host cell invasion." PLoS One **9**(3): e91819.

Whitelaw, J. A., F. Latorre-Barragan, S. Gras, G. S. Pall, J. M. Leung, A. Heaslip, S. Egarter, N. Andenmatten, S. R. Nelson, D. M. Warshaw, G. E. Ward and M. Meissner (2017). "Surface attachment, promoted by the actomyosin system of Toxoplasma gondii is important for efficient gliding motility and invasion." BMC Biol **15**(1): 1.
