# Supplementary material for: Formin-2 drives polymerisation of actin filaments enabling segregation of apicoplasts and cytokinesis in Plasmodium falciparum
Source: eLife. 2019 Jul 19;8:e49030. doi: 10.7554/eLife.49030 (PMC6688858; doi:10.7554/eLife.49030)
Supplement: Supplementary file 1. [file elife-49030-supp1.docx]

**Supplementary File 1. Antibodies used in this study**

| **Antibody** | **Reference** |
| --- | --- |
| Anti-actin1 | (Angrisano, Riglar et al. 2012), RRID: AB_2665920 |
| Anti-CPN60 (apicoplast) | (Agrawal, van Dooren et al. 2009) |
| Anti-RON4 | (Richard, MacRaild et al. 2010) |
| Anti-GAP45 | (Jones, Kitson et al. 2006) |
| Anti-MTIP | (Jones, Kitson et al. 2006) |
| Anti-Enolase | (Dutta, Tewari et al. 2018) |
| Anti-HA | Roche (Cat #118743100) |
| Anti-YFP | Abcam (Cat #ab6556) |
| Anti-Atrx1 | (DeRocher, Coppens et al. 2008) |
| Anti-G2Trx | Biddau and Sheiner, unpublished. |
| Anti-TOM40 | (van Dooren, Yeoh et al. 2016), (Ovciarikova, Lemgruber et al. 2017) |

Agrawal, S., G. G. van Dooren, W. L. Beatty and B. Striepen (2009). "Genetic evidence that an endosymbiont-derived endoplasmic reticulum-associated protein degradation (ERAD) system functions in import of apicoplast proteins." J Biol Chem **284**(48): 33683-33691.

Angrisano, F., D. T. Riglar, A. Sturm, J. C. Volz, M. J. Delves, E. S. Zuccala, L. Turnbull, C. Dekiwadia, M. A. Olshina, D. S. Marapana, W. Wong, V. Mollard, C. H. Bradin, C. J. Tonkin, P. W. Gunning, S. A. Ralph, C. B. Whitchurch, R. E. Sinden, A. F. Cowman, G. I. McFadden and J. Baum (2012). "Spatial localisation of actin filaments across developmental stages of the malaria parasite." PLoS One **7**(2): e32188.

DeRocher, A. E., I. Coppens, A. Karnataki, L. A. Gilbert, M. E. Rome, J. E. Feagin, P. J. Bradley and M. Parsons (2008). "A thioredoxin family protein of the apicoplast periphery identifies abundant candidate transport vesicles in Toxoplasma gondii." Eukaryot Cell **7**(9): 1518-1529.

Dutta, S., A. Tewari, C. Balaji, R. Verma, A. Moitra, M. Yadav, P. Agrawal, D. Sahal and G. K. Jarori (2018). "Strain-transcending neutralization of malaria parasite by antibodies against Plasmodium falciparum enolase." Malar J **17**(1): 304.

Jones, M. L., E. L. Kitson and J. C. Rayner (2006). "Plasmodium falciparum erythrocyte invasion: a conserved myosin associated complex." Mol Biochem Parasitol **147**(1): 74-84.

Ovciarikova, J., L. Lemgruber, K. L. Stilger, W. J. Sullivan and L. Sheiner (2017). "Mitochondrial behaviour throughout the lytic cycle of Toxoplasma gondii." Sci Rep **7**: 42746.

Richard, D., C. A. MacRaild, D. T. Riglar, J. A. Chan, M. Foley, J. Baum, S. A. Ralph, R. S. Norton and A. F. Cowman (2010). "Interaction between Plasmodium falciparum apical membrane antigen 1 and the rhoptry neck protein complex defines a key step in the erythrocyte invasion process of malaria parasites." J Biol Chem **285**(19): 14815-14822.

van Dooren, G. G., L. M. Yeoh, B. Striepen and G. I. McFadden (2016). "The Import of Proteins into the Mitochondrion of Toxoplasma gondii." J Biol Chem **291**(37): 19335-19350.
