# Supplementary material for: Formin-2 drives polymerisation of actin filaments enabling segregation of apicoplasts and cytokinesis in Plasmodium falciparum
Source: eLife. 2019 Jul 19;8:e49030. doi: 10.7554/eLife.49030 (PMC6688858; doi:10.7554/eLife.49030)
Supplement: Supplementary file 2. [file elife-49030-supp2.docx]

**Supplementary File 2. List of UniProt proteomes used**

{up000033188}Babesia bigemina                        BABBI
{up000199752}Cryptosporidium hominis                CRYHO
{up000001460}Cryptosporidium muris RN66                CRYMR
{up000006726}Cryptosporidium parvum Iowa II            CRYPI
{up000095192}Cyclospora cayetanensis                9EIME
{up000030754}Eimeria tenella                        EIMTE
{up000019763}Gregarina niphandrodes                    GRENI
{up000027470}Hammondia hammondi                        HAMHA
{up000008983}Ichthyophthirius multifiliis            ICHMG
{up000007494}Neospora caninum Liverpool                NEOCL
{up000000600}Paramecium tetraurelia                    PARTE
{up000007800}Perkinsus marinus ATCC 50983            PERM5
{up000074855}Plasmodium berghei ANKA                PLABA
{up000071118}Plasmodium chabaudi chabaudi            PLACH
{up000006319}Plasmodium cynomolgi strain B            9APIC
{up000001450}Plasmodium falciparum 3D7                PLAF7
{up000054561}Plasmodium fragile                        PLAFR
{up000076004}Plasmodium gaboni                        9APIC
{up000030640}Plasmodium inui San Antonio 1            9APIC
{up000031513}Plasmodium knowlesi strain H            PLAKH
{up000078597}Plasmodium malariae                    PLAMA
{up000078550}Plasmodium ovale wallikeri             9APIC
{up000030659}Plasmodium vinckei peterei                PLAVN
{up000009168}Tetrahymena thermophila SB210            TETTS
{up000001950}Theileria annulata                        THEAN
{up000031512}Theileria equi strain WA                THEEQ
{up000003786}Theileria orientalis strain Shintoku    THEOR
{up000001949}Theileria parva                        THEPA
{up000002226}Toxoplasma gondii                      TOXGV
{up000002195}Dictyostelium discoideum AX4            DICDI
{up000001548}Giardia lamblia ATCC 50803                GIAIC
{up000008524}Trypanosoma brucei brucei TREU927        TRYB2
{up000001449}Thalassiosira pseudonana                THAPS
{up000006548}Arabidopsis thaliana (thale cress)        ARATH
{up000007014}Cyanidioschyzon merolae                CYAM1
{up000005640}Homo sapiens (human)                    HUMAN
